# Keratoacanthoma-like eruption associated with pembrolizumab and dupilumab treated by intralesional triamcinolone: A case report

**DOI:** 10.1016/j.jdcr.2025.03.036

**Published:** 2025-04-26

**Authors:** Raphaella Lambert, Elizabeth Rotrosen, Hannah Verma, Grace Rabinowitz, Patricia Heller, Nicholas Gulati

**Affiliations:** Kimberly and Eric J. Waldman Department of Dermatology, Icahn School of Medicine at Mount Sinai, New York, New York

**Keywords:** cutaneous immune-related adverse event, dupilumab, immunotherapy, keratoacanthoma, lichenoid, pembrolizumab

## Introduction

Pembrolizumab is an immune checkpoint inhibitor (ICI) associated with various cutaneous immune-related adverse events (cirAEs), which can occur even after ICI discontinuation.^1^ Dupilumab, a monoclonal antibody against interleukin (IL)-4 and IL-13, is used in the management of cirAEs.^2^ Although rare, squamoproliferative and keratoacanthoma-like eruptions (KLEs) have been reported in the setting of pembrolizumab and dupilumab therapy.^3^, ^4^, ^5^ We present a case of a KLE in a patient previously treated with pembrolizumab and on current dupilumab therapy, resolving with intralesional triamcinolone (ILTAC) injection.

## Case report

An 86-year-old man with metastatic prostate cancer presented to the dermatology clinic for evaluation of an erosive, painful, and pruritic rash on the extremities, trunk, and lips, present for 6 months. The patient’s cancer had been treated with 6 cycles of pembrolizumab between June and October 2023 and was discontinued after an episode of diabetic ketoacidosis and new-onset diabetes; he remained on leuprolide therapy. The rash appeared in November 2023, and he was initially diagnosed with guttate psoriasis by an outside dermatologist and prescribed clobetasol 0.05% ointment, to which he did not respond after several weeks of twice daily use. He was re-evaluated in January 2024 and prescribed calcipotriene, oral fluconazole, and ciclopirox cream for suspected intertrigo. Later that month, he was seen by another outside dermatologist, who performed 3 biopsies, all demonstrating patchy lichenoid lymphocytic infiltrate with scattered eosinophils and keratinocyte necrosis, consistent with a lichenoid drug reaction. He was prescribed triamcinolone 0.1% ointment, which he used twice daily for several months without improvement.

**Fig 1:**
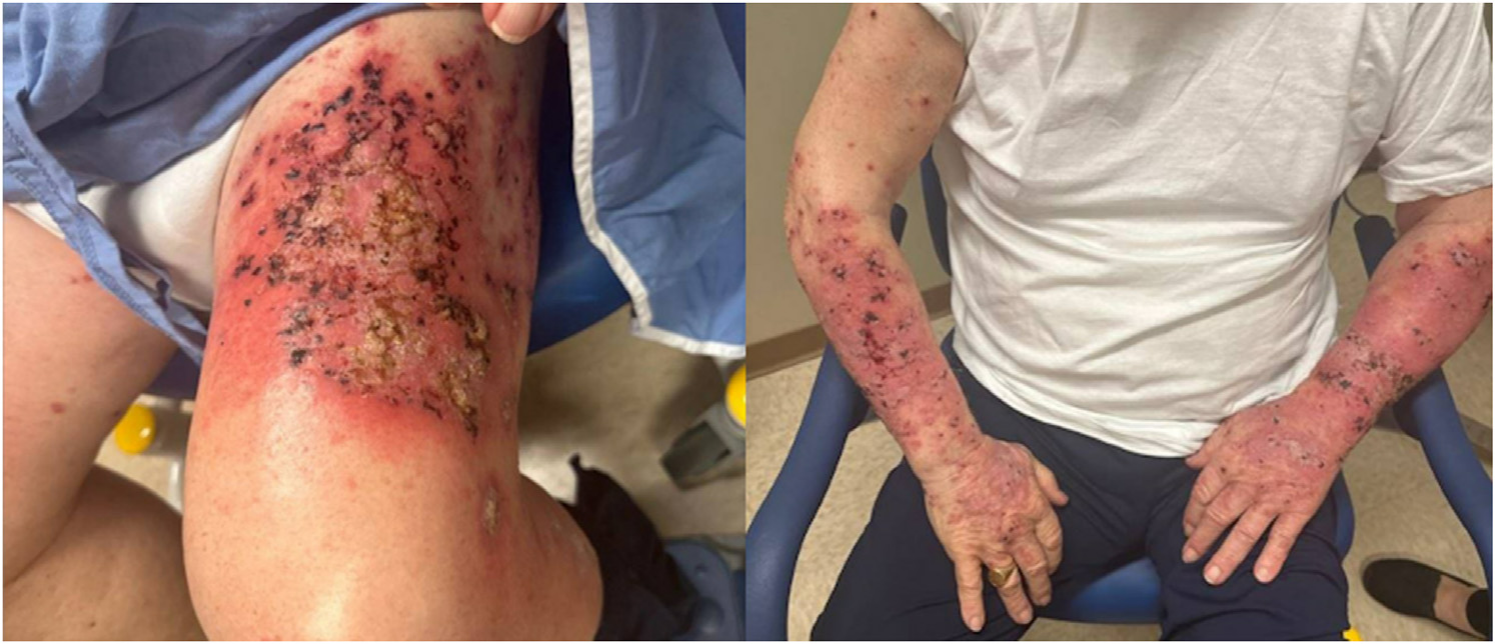
Baseline biopsy-proven lichenoid rash (May 2024).

On initial presentation to our clinic in May 2024, crusted, ulcerated erythematous papules and plaques were noted on the bilateral upper and lower extremities, abdomen, and cutaneous lower lip (Fig 1). Few small, intact bullae were observed. The patient had no mucosal involvement and Nikolsky sign was absent. Total body surface area involvement was 50%, with an Investigator Global Assessment score of 4.

Given the severity and extent of the patient’s disease and the failure of topical therapies, dupilumab was recommended as an off-label treatment for lichenoid cirAE (600 mg loading dose followed by 300 mg subcutaneously every 2 weeks).

**Fig 2:**
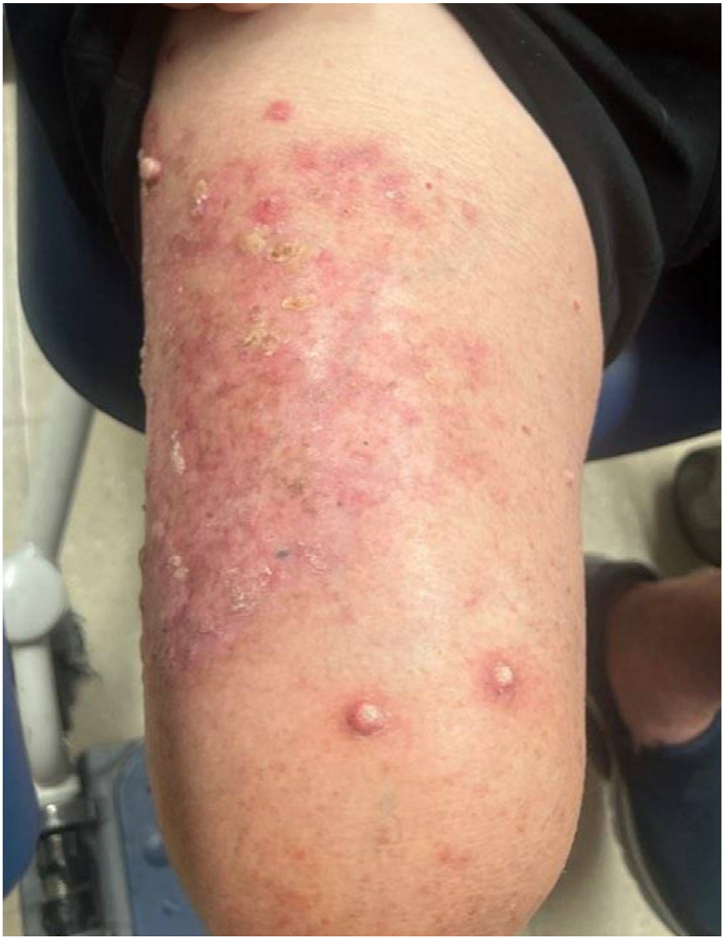
Erythematous nodules on the right thigh, biopsy-proven as squamous cell carcinoma, keratoacanthoma type (July 2024).

Four weeks later (June 2024), he reported improvement in his rash and pruritus after starting dupilumab. Six weeks later (July 2024), his lichenoid rash continued to improve, but his wife described the emergence of “horns” on the patient’s right thigh, developing over the last month. Examination revealed 3 erythematous nodules with central crust on the right thigh (Fig 2). All lesions were biopsied, with pathology for each site demonstrating atypical squamous cells in the dermis, consistent with squamous cell carcinoma, keratoacanthoma type.

**Fig 3:**
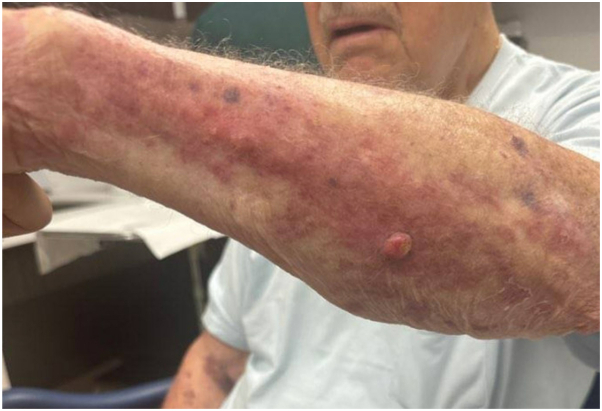
Probable squamous cell carcinoma, keratoacanthoma type, on the left forearm before treatment (August 2024).

Treatment options for the biopsy-confirmed squamous cell carcinomas were discussed, including excision and ILTAC injection. The patient declined treatment, as the lesions were asymptomatic. In August 2024, he reported a new, similar lesion on the left forearm that was causing pain and bleeding. Clinical examination was consistent with another probable squamous cell carcinoma, keratoacanthoma type (Fig 3). Given his various comorbidities, the patient elected for treatment with ILTAC, and 0.2 cc of ILTAC 40 mg/cc was administered to the arm lesion.

**Fig 4:**
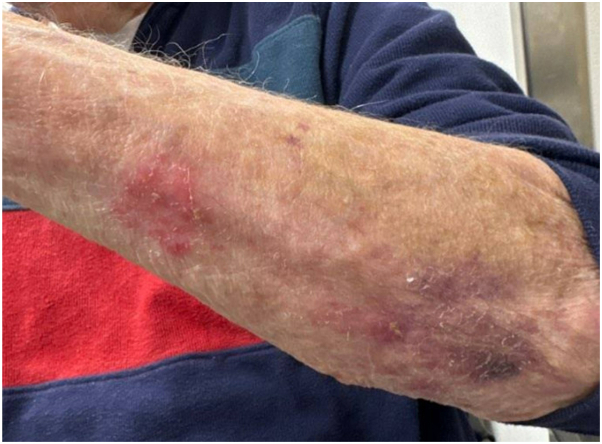
Probable squamous cell carcinoma, keratoacanthoma type resolved after 2 rounds of intralesional triamcinolone (December 2024).

By September 2024, the lesion on his left forearm had slightly grown. The decision was made to retreat the lesion with 0.3 cc of ILTAC 40 mg/cc. In October 2024, he reported less pain and pruritus of the arm lesion, although the size was unchanged. He declined further treatment at that time. At his most recent visit in December 2024, the arm lesion had resolved; the leg lesions have remained clinically resolved since their initial biopsies, which removed the entirety of the visible lesions (Fig 4). He has continued dupilumab therapy, with significant improvement in his lichenoid rash, and no development of any further keratoacanthomatous lesions.

## Discussion

KLEs have been observed with several ICIs, including pembrolizumab and nivolumab,^3^^,^^4^^,^^6^ with eruptions typically appearing 2 to 18 months after initiation of immunotherapy in patients with a median age of 80 years.^3^ The exact mechanisms underlying the development of KLEs among ICI patients remains unclear; anti-programmed cell death protein 1 therapies may foster an immunological response against subclinical epidermal UV-induced dysplasia, resulting in transient epithelial proliferation.^3^ The advanced age of patients with KLE and the location of most eruptions on chronically UV-exposed skin support this hypothesis.^3^ KLEs have also been observed in areas of resolving, concomitant, or delayed lichenoid reactions,^4^ as in our case. The mechanisms driving the development of KLEs or other secondary cutaneous neoplasms in patients on ICIs may be similar to those causing tumor pseudoprogression that has been associated with these medications^3^^,^^7^; however, further research is warranted. Although dupilumab is largely considered to be immunomodulatory and not immunosuppressive, it is possible that IL-4 and/or IL-13 blockade in a patient previously on an ICI contributed to the rapid development of a KLE. IL-4 induces the production of transforming growth factor β, which functions as a cell cycle regulator; as such, dupilumab decreases transforming growth factor β production, which in turn reduces control of cell cycle proliferation, potentially contributing to epithelial proliferation.^5^

Optimal management of KLEs remains controversial, given the morbidity associated with both surgical and medical options,^8^ as well as the questionable malignant potential of these lesions.^4^^,^^7^^,^^9^ In fact, some authors prefer the term eruptive squamous atypia to describe this entity, given that many lesions in this disease fail to meet clinical and histologic criteria for true keratoacanthomas.^9^ Although surgical excision may be recommended for solitary nodules, first-line therapies for eruptive lesions include systemic retinoids, alone or in combination with excision.^8^ Other treatments including cyclosporine, intralesional steroids, methotrexate, or 5-fluorouracil, topical steroids, curettage, or cryosurgery have been reported.^8^^,^^9^ Given our patient’s history of poor wound healing and extensive comorbidities, the decision was jointly made to pursue a minimally invasive, localized approach with ILTAC, to which the arm lesion ultimately responded. The resolution of this lesion with localized, anti-inflammatory therapy may further support research suggesting that eruptive keratoacanthomas in patients on immunotherapy represent reversible hyperplasia rather than true malignancy.^4^

We highlight this case to add to the literature on pembrolizumab-induced cirAEs and more specifically, keratoacanthoma-like lesions. Further research into the development and treatment of such lesions among patients exposed to immunotherapy and/or dupilumab is warranted.

## Conflicts of interest

None disclosed.
